# RETRACTION: Meta‐Analysis on the Impact of Immune Senescence: Unravelling the Interplay in Cutaneous Wound Healing and Lung Cancer Progression

**DOI:** 10.1111/iwj.70531

**Published:** 2025-04-18

## Abstract

**RETRACTION:** DongM. , JinY. , LinX. , WangS. , and ShenP. , “Meta‐Analysis on the Impact of Immune Senescence: Unravelling the Interplay in Cutaneous Wound Healing and Lung Cancer Progression,” International Wound Journal 21, no. 2 (2024): e14756, 10.1111/iwj.14756.38339818 PMC10858335

The above article, published online on 09 February 2024, in Wiley Online Library (http://onlinelibrary.wiley.com/), has been retracted by agreement between the journal Editor in Chief, Professor Keith Harding; and John Wiley & Sons Ltd. Following an investigation by the publisher, all parties have concluded that this article was accepted solely on the basis of a compromised peer review process. The editors have therefore decided to retract the article. The authors did not respond to our notice regarding the retraction.

**RETRACTION:** M. Dong , Y. Jin , X. Lin , S. Wang , and P. Shen , “Meta‐Analysis on the Impact of Immune Senescence: Unravelling the Interplay in Cutaneous Wound Healing and Lung Cancer Progression,” International Wound Journal 21, no. 2 (2024): e14756, 10.1111/iwj.14756.38339818 PMC10858335

The above article, published online on 09 February 2024, in Wiley Online Library (http://onlinelibrary.wiley.com/), has been retracted by agreement between the journal Editor in Chief, Professor Keith Harding; and John Wiley & Sons Ltd. Following an investigation by the publisher, all parties have concluded that this article was accepted solely on the basis of a compromised peer review process. The editors have therefore decided to retract the article. The authors did not respond to our notice regarding the retraction.

